# The power of children’s sleep - Improved declarative memory consolidation in children compared with adults

**DOI:** 10.1038/s41598-020-66880-3

**Published:** 2020-06-19

**Authors:** Anna Peiffer, Maud Brichet, Xavier De Tiège, Philippe Peigneux, Charline Urbain

**Affiliations:** 1 Laboratoire de Cartographie fonctionnelle du Cerveau (LCFC), UNI – ULB Neuroscience Institute, Université libre de Bruxelles (ULB), Brussels, Belgium; 2 Neuropsychology and Functional Imaging Research Group (UR2NF), Center for Research in Cognition and Neurosciences (CRCN), UNI – ULB Neuroscience Institute, Université libre de Bruxelles (ULB), Brussels, Belgium

**Keywords:** Circadian rhythms and sleep, Learning and memory

## Abstract

Post-learning slow wave sleep (SWS) is known to support declarative memory consolidation. As SWS is more abundant in young population, we suggested that sleep-dependent memory consolidation processes could occur at a faster pace in school-aged children. After learning new associations between non-objects and their functions, retrieval performance was tested in 30 children (7–12 years) and 34 adults (20–30 years) during an immediate (IR) and a delayed retrieval (DR) session separated by either a Sleep or a Wake condition. Sleep led to stabilized memory retrieval performance only in children, not in adults, whereas no age-related difference was observed after a similar period of wakefulness. Hence, our results suggest more efficient sleep-dependent declarative memory consolidation processes in children compared with adults, an effect potentially ascribed to more abundant and deeper SWS during childhood.

## Introduction

Children, particularly at school-age, have to learn and consolidate high quantities of new declarative information (e.g. learning a vocabulary list in a foreign language) to respond adequately to environmental demands. According to system consolidation theories, declarative knowledgeis progressively integrated into long-term memory through brain plasticity processes generating functional and structural changes at the neural level^1,2^. Both in adults^3–8^ and across development^9^, studies showed that sleep plays an active role in these plasticity-related changes promoting memory consolidation processes. In particular, slow wave sleep (SWS) has been suggested to trigger (i.e. elicit) the transfer of newly learned representations, initially stored in the hippocampal and para-hippocampal areas, towards prefrontal brain areas for long term storage^8,10–12^. Childhood, compared with adulthood, is not only characterized by a higher amount and variety of learning experiences supported by cerebral plasticity processes^13,14^ but also by a higher amount of SWS^15–18^. Compelling evidence suggests that children (7–12 years old) spend significantly longer proportion of their night sleep time in SWS (around 25 to 35%) than adults (around 15 to 20%)^15,18–21^. As several studies suggested that SWS markedly contributes to declarative memory consolidation processes across development and as SWS is more abundant in school-age children than adults, it has been suggested that sleep-dependent memory consolidation processes may be more efficient and/or accelerated in children than in adults^22,23^. In line with this proposal, it has been shown using magnetoencephalography (MEG) that in 7–11 year old children, a 90-minute daytime nap is already sufficient to trigger changes in signal amplitude of the neural substrates related to the long-term storage of newly learned declarative material^23^. Importantly, a similar brain reorganization associated with the course of memory consolidation was previously observed in adults, but only days to months after the initial learning session^8,12^.

At the behavioural level, the beneficial impact of sleep on declarative memory consolidation has been observed in adults^3,5,8,22,24^, children^22,25–27^, and adolescents^28^ either through stabilized or improved memory retention performance at delayed recall (compared with immediate recall). Yet, to the best of our knowledge, only three studies have compared the impact of sleep on memory consolidation performance between children and adults^22,29,30^ and did not highlight a developmental advantage of sleep on memory consolidation performance. For instance, Wilhelm *et al*. (2008)^22^ compared sleep-dependent declarative memory consolidation performance between children aged 6–8 years and adults, using a classical word-pair associate learning task. Participants had a better recall performance after a night of sleep compared to a similar period of wakefulness. However, overnight improvements in performance were similar between adults and children, despite a twice as large amount of SWS in children than in adults during the post-training sleep period. Since pre-existing knowledge boosts memory consolidation processes^31–33^, the authors explained this lack of effect by the imbalance of pre-existing representations associated with the learning material between children and adults. Smaller amount of schemata and knowledge associated to the newly learned material (i.e., word pairs) in children may have prevented observing a potential advantage of sleep on memory consolidation performance in the younger population compared with adults^22,34^.

In this framework, the present study investigated the potential age-related advantage of sleep on declarative memory consolidation performance using a learning task that allows a clear comparison between children and adults, by minimizing the impact of pre-existing representations on the to-be-learned material. To do so, we explored the impact of sleep on the consolidation of new associations between non-objects and their “magical” function, a material that is equally novel for adults and children^23,35^. We hypothesize that after controlling for the impact of pre-existing representations, children (7–12 years) would exhibit larger gains in memory retention performance (i.e., difference between the number of correctly recalled items at the delayed vs. the immediate retrieval sessions) than adults (20–30 years) over a night of sleep.

## Results

### Sleep and circadian parameters

Sleep parameters are reported in Table 1.

**Table 1:** Sleep parameters.

	Sleep		Wake	
	Children	Adults	Children	Adults
Duration <6 months (h)	9.43 ± 0.42	8.09 ± 0.92	9.7 ± 0.75	7.92 ± 1.03
Duration N-2 (h)	9.93 ± 1.25	9.03 ± 1.35	10.10 ± 0.99	8.79 ± 1.31
Duration N-1 (h)	10.12 ± 1.37	8.20 ± 1.08	10.37 ± 1.22	8.42 ± 0.69
Duration N-0 (h)	9.27 ± 1.00	7.53 ± 0.78	—	—
Onset <6 months (min)	20.00 ± 7.56	21.88 ± 19.65	19.33 ± 8.00	19.28 ± 12.58
Onset N-2 (min)	18.00 ± 17.09	21.38 ± 24.28	17.13 ± 12.32	24.28 ± 28.89
Onset N-1 (min)	22.00 ± 16.01	24.06 ± 28.89	14.67 ± 13.43	16.39 ± 13.92
Onset N-0 (min)	10.00 ± 9.45	32.81 ± 39.20	—	—

Mean (±SEM) amount of sleeping hours (duration) and the latency (minutes) required to fall asleep (onset) for the last months, the two nights before the experiment (N-2 and N-1), and the night between the experiment and the delayed retrieval session (N-0).

Sleep duration did not differ between the Sleep and Wake groups (see Methods section) during the nights preceding the experiment (Night-2, Sleep: 9.93 ± 1.25 vs. Wake: 10.03 ± 0.88 h; Night-1, Sleep: 10.12 ± 1.37 vs. Wake: 10.38 ± 1.21 h), either in the children (all *p*s > 0.18) or in the adult (all *p*s > 0.25) groups.

In children, the average sleep duration for the two nights preceding the experiment did not differ from their average sleep time over the past month (N-2: mean ± SD 10.0 ± 1.1 h, *p* = 0.5; N-1: 10.2 ± 1.3 h, *p* = 0.015). Although adults slept longer during N-2 than they did on average over the past six months, the average sleep duration of N-1 did not differ from the average sleep time in the past six months (N-2: mean ± SD 8.9 ± 1.3 h, *p* = 0.001; N-1: 8.3 ± 0.9 h; *p* = 0.17).

During the post-learning night (N-0), sleep duration did not differ from the average sleep duration in the past six months, either for children (N-0: mean ± SD 9.3 ± 1.0 h, *p* = 0.62) or adults (N-0: mean ± SD 7.5 ± 0.8 h, *p* = 0.11). However, on average children slept significantly more than adults (*p* = 0.001).

### Vigilance parameters

Vigilance parameters are reported in Table 2.

**Table 2:** Vigilance parameters.

	Sleep		Wake	
	Children	Adults	Children	Adults
Vigilance Index (ms)	−7.20 ± 40.67	−10.19 ± 16.70	2.88 ± 30.32	−5.34 ± 16.37
PVT session 1 (ms)	460 ± 87.66	341.25 ± 31.18	425.07 ± 66.72	352.94 ± 29.21
PVT session 2 (ms)	452 ± 85.48	331.06 ± 29.43	424.87 ± 55.02	347.61 ± 29.14

Psychomotor vigilance task (PVT). Mean (± SEM) for the vigilance index (ms) obtained by subtracting mean reaction times (RTs) in the delayed session from mean RTs in the immediate session (ms), PVT mean RTs at the first session (1) and PVT mean RTs at the second session (2).

A repeated measures ANOVA analysis was conducted on Reaction Times (RTs) with one within-subject factor SESSION (immediate retrieval, IR vs. delayed retrieval, DR) and two between-subjects factors CONDITION (Sleep vs. Wake) and AGE GROUP (Children vs. Adults). Results did not show any main effect of session or of condition nor interaction effect between these factors (all *p*s > 0.10). A main effect of age group [F_1,60_ = 52.32; *p* = 0.00001; η^2^ = 0,465] was observed, with faster RTs in adults than in children (Children: mean ± SD 440.8 ± 74.8 ms; Adults: 343.6 ± 30.2 ms). Hence, vigilance performance remained stable between both retrieval sessions in both conditions for children and for adults.

### Memory retention performance: age-related and sleep effects

**Figure 1:**
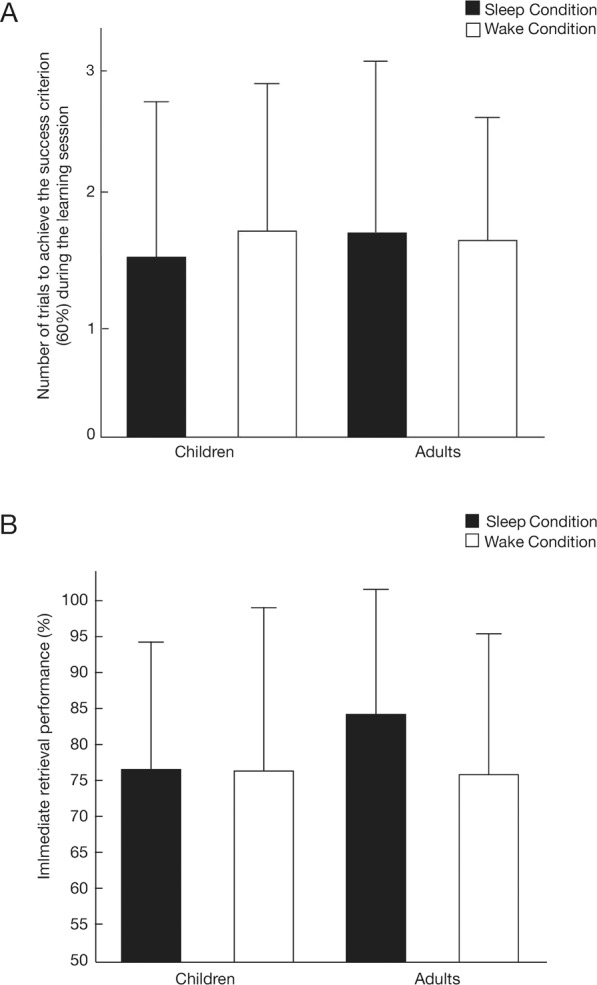
Learning performance (mean ± s.e.m.). (**A**) Number of trials to achieve the success criterion of 60% for children (N = 15) and adults (N = 16) in the Sleep condition and for children (N = 15) and adults (N = 18) in the Wake condition. (**B**) Immediate retention performance (% of correct responses for the immediate retrieval session occurring directly after the learning session) for children (N = 15) and adults (N = 16) in the Sleep condition and for children (N = 15) and adults (N = 18) in the Wake condition.

A t-test for independent groups showed that the number of trials needed to reach the successful criterion (60% of correct responses) in the learning session did not differ between children and adults (Children: mean ± SD trials 1.57 ± 0.68 vs. Adults: 1.65 ± 0.54, *p* = 0.6, see Fig. 1A). A Bayesian t-test for independent groups also supported the null hypothesis (BF = 0.113), indicating that the learning task difficulty was comparable between children and adults. Likewise, a factorial ANOVA analysis conducted on IR performance with between-subjects factors AGE GROUP (Children vs. Adults) and CONDITION (Sleep vs. Wake) did not show any main effect of AGE GROUP [F_1,60_ = 2.11; *p* = 0.15; η^2^ = 0.034] or CONDITION [F_1,60_ = 3.18; *p* = 0.08; η^2^ = 0.050], nor an interaction effect [F_1,60_ = 2.61; *p* = 0.11; η^2^ = 0.042], suggesting a comparable task difficulty between age groups and conditions (see Fig. 1B).

Descriptive analyses performed on retention indices (i.e., % correct responses at the DR – IR) showed that, after a night of sleep, children successfully recalled 1.87 ± 4.86% more definitions at DR in comparison with IR. After the same night of sleep, adults forgot on average 4.75 ± 4.84% of the definitions they were able to retrieve in the evening the day before (IR session). In the Wake condition, both age groups forgot a similar amount of learned information between the DR and the IR sessions (Children: −2.53 ± 2.56% ; Adults: − 4.67 ± 3.56%).

**Figure 2:**
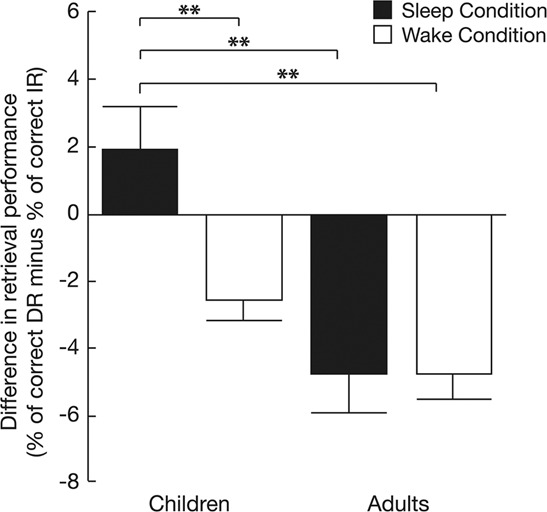
Memory retention performance (mean ± s.e.m). Retention indices (percentage of correct responses at delayed retrieval minus percentage of correct responses at immediate retrieval) in children (N = 15) and adults (N = 16) in the Sleep condition and in children (N = 15) and adults (N = 18) in the Wake condition. Asterisks indicate a significant difference between age groups (children VS adults): *p ≤ 0.05 or **p ≤ 0.01.

To statistically probe the effect of age on sleep-dependent memory consolidation, a factorial ANOVA analysis conducted on the retention indices was performed with two between-subjects factors, namely AGE GROUP (Children vs. Adults) and CONDITION (Sleep vs. Wake). Results showed a main effect of AGE GROUP [F_1,60_ = 18.45; *p* = 0.001; η^2^ = 0.235] and CONDITION [F_1,60_ = 4.49; *p* = 0.04; η^2^ = 0.070] as well as a significant AGE GROUP by CONDITION interaction [F_1,60_ = 4.85; *p* = 0.03; η^2^ = 0.075]. Post-Hoc Tukey tests showed that in the Sleep condition, children had higher retention indices than adults (*p* = 0.00003), whereas retention indices did not differ between age groups in the Wake condition (*p* = 0.06) (see Fig. 2). The Bayesian t-test with AGE GROUP as the between-subjects factor supported the hypothesis in the Sleep condition (BF = 4.36; *p* = 0.001) and was not conclusive in the Wake condition (BF = 0.76).

Finally, correlation analyses showed that sleep duration and sleep onset on the experiment night were not correlated with the retention indices (Sleep duration: Pearson correlation (2-tailed) r = 0.164; *p* = 0.38; Sleep onset: Pearson correlation (2-tailed) r = −0.05; *p* = 0.80) (Fig. 2).

## Discussion

This study demonstrates stronger overnight gains of declarative memory retrieval performance in children compared with adults. Our results showed that after a night of sleep, in children, memory retention performance was stabilized (i.e., there was no memory loss, reflecting successful consolidation^22^) between the immediate and delayed retrieval sessions while memory retrieval performance decreased in adults. Importantly, delayed retrieval performance decreased both in children and adults after an equivalent interval of wakefulness, strengthening the specific effect of sleep on the age-related differences observed in our study.

Given the higher proportion of SWS in children than adults and considering the crucial role of this sleep stage for memory consolidation^9,15,18^, several authors hypothesized a developmental advantage of sleep for memory consolidation processes in children^22,23,34^. Highly consistent with this hypothesis, our results are surprisingly the first to show this effect. Previous studies did not highlight improved sleep-dependent memory consolidation performance in children compared with adults^22,30^. In these studies, the lack of age-related differences on sleep-dependent memory consolidation performance was explained by potential unbalanced learning-related pre-existing representations between children and adults^22,34^. Reinforcing this idea, animal studies showed that larger schemas in long-term memory boost memory consolidation processes^31,32^.

This study addressed this issue by using a learning material that was equally novel for adults and children. This allowed us to accurately compare age-related differences of sleep on memory consolidation performance. Results confirmed that both children and adults showed equivalent learning performance at the immediate retrieval session and needed a similar number of trials to reach the successful learning criterion (60%). Thus, contrary to previous studies, our results cannot be explained by an unbalanced task difficulty between adults and children. Sleep parameters and vigilance performance were also carefully controlled at each retrieval session in both age groups. We observed comparable vigilance performance between retrieval sessions (IR vs. DR) and experimental conditions (Sleep vs. Wake) in children and adults. These observations excluded a potential contribution of circadian or vigilance effects on the observed sleep-dependent differences between age groups. Sleep parameters were also assessed by self-reported questionnaires and were equivalent between both age-groups apart from the number of hours of sleep which was, as expected, higher in children than in adults. Correlations between hours of sleep and retention performance did not reach significance, similarly to previous findings suggesting that retention performance may not be dependent on the total amount of sleep, but more specifically on SWS rates^36^.

SWS has been related to hippocampal activation^37^ and hippocampus-dependent memory consolidation processes^6^. Related to a dialogue between neocortical and (para)-hippocampal areas, it has been hypothesized that SWS would trigger better memory consolidation by transferring new information from the hippocampus and the para-hippocampus, the intermediate storage areas, to the neocortex for long-term storage and integration into memory networks^8,10,34^. Remarkably, up to pubertal age, children show more SWS^15,16^ than adults. In this respect, our results provide additional support for the crucial role of sleep for memory consolidation processes during childhood. Our findings suggest that age-related differences in memory retrieval performance specifically observed after a night of sleep are related to a beneficial role of SWS for children compared with adults. Future neurophysiological studies are needed to confirm this hypothesis.

We expected children to benefit more from sleep than adults to consolidate memories, a prediction confirmed by our data. However, an overnight decrease of performance in adults was unexpected, since several studies showed a benefit of sleep on declarative memory consolidation performance in adults^8,22,26^. One explanation could be that, in adults, totally novel representations are poorly consolidated by post-training sleep, as prior knowledge seems to be a prerequisite for new memories to be consolidated during sleep^38–40^. In that line, studies in rodents^31,32^ have highlighted a strong impact of retroactive interference on totally new representations. New information related to pre-existing knowledge seems less sensitive to retroactive interference, and therefore features a slower deterioration rate. Moreover, the declarative memory network contribution to successful memory retrieval is enhanced for the schema-congruent relative to schema-incongruent memories in adults^39,40^. Interestingly, Brod *et al*. (2017) showed that hippocampal and neocortical contributions during a retrieval session were not dependent on pre-existing knowledge in children. However, in adults, activation of the declarative memory networks significantly depended on the congruence of the new information with prior learning^41^. Hence, in line with these previous studies, our results suggest that pre-existing schemas wield a specific effect on memory consolidation processes in adults, which does not seem to be the case in children.

In conclusion, the present study supports the importance of sleep and its impact on learning processes during childhood. By comparing sleep-dependent memory consolidation performance between children and adults differing on the amount of SWS^15,16,18^, and by showing the specific advantage of sleep in children on memory consolidation performance, our experiment presumed to advance the study of brain mechanisms underlying age-related changes in sleep-dependent memory consolidation processes. Hence, our findings open up novel avenues to investigate how age-related changes in SWS-dependent memory consolidation processes may be related to different underlying neurophysiological processes in children and adults both in the context of typical or atypical developmental conditions.

### Limitation section

We acknowledge limitations in the present study that could be addressed in the future. First, we did not record electrophysiological sleep parameters and therefore could not quantify SWS in our young and adult populations, or search for potential correlations with overnight changes in memory performance. However, we believe that it does not hamper the validity of our main conclusions based on the known differences in SWS between prepubertal and adult populations. Over the past 15 years, developmental studies have consistently demonstrated that the amount of SWS drastically decreases with age across the maturational process (see Kurdziel, 2019^9^, for a recent and detailed review on this topic). From 6 years old to prepubertal age, the SWS rate as well as its different characteristics are quite similar despite age differences^17,18^. Across puberty, SWS rate sharply declines in association with a decrease of cortical connectivity resulting from brain maturation and reduced synaptic density^15,19,20^ and continue to decrease with age^16,18^. For instance, Kurth *et al*. (2010) showed that while prepubertal children (≤12 years old) exhibited a SWS rate of 28.1 (±2.8) % per night, mature adolescents already showed a significant decrease in SWS rate with 19.3 (±1.7) % per night^21^. Thus, according to these numerous developmental studies and, as we compared school-age children (7–12 years) and adults (20–30 years), we assume that SWS durations per night differ significantly between populations without overlap in our study. We also chose not to obtain EEG recordings in this study, to have children and adults sleeping in more natural conditions.

Second, due to the absence of interference in the sleep condition, one may hypothesize that sleep may act as a “temporary shelter” that simply postpones the effect of interference and, thereby, passively maintains the memory traces. However, compelling evidence have critically challenged this view and showed that sleep-dependent gains in memory performance do not solely result on the basis of reduced interference but depend on an active role of sleep. At the behavioural level, Ellenbogen and coll. demonstrated in two important behavioural studies not only that sleep improves recall of verbal memories despite the presence of retroactive interference but also that sleep renders these newly formed memories more resistant, especially when it is challenged with interference^42,43^. In addition to these studies, a great body of neurophysiological studies also demonstrate the active role of sleep in consolidating memory. In particular, several studies showed, using polysomnographic recordings, that sleep-dependent improvements in performance were associated with a specific sleep stage (e.g. SWS but not REM sleep or Sleep stage 2)^44–47^. Furthermore, neuroimaging studies conducted in adults showed reactivation of learning-related cerebral areas during sleep and that the amplitude of these activations were correlated with the amount of overnight gains in performance^6,48^. Altogether, these studies demonstrated that sleep plays an active role in declarative memory consolidation processes, thus, rejecting critics asserting that the beneficial impact of sleep on memory performance results from a passive and temporary protection against interference that would otherwise be observed during wakefulness.

For future directions, we believe that further studies should investigate changes in sleep-dependent memory consolidation processes across various developmental age groups, including younger children (<7 years old) and adolescents (12–18 years old). This may also help better understand how sleep-dependent memory consolidation changes may differentially impact academic performance and general cognitive abilities. In particular, comparing these aspects before and after adolescence should provide us with important information, as a substantial decrease in the SWS rate (for a similar night of sleep) occurs during this developmental period^15,21,49–51^. We also suggest future work to investigate the link between sleep disorders and learning disabilities, as evidence shows an association between atypical sleep patterns in children (e.g., interictal epileptic activity during slow sleep) and memory difficulties^23,52–56^.

## Methods

### Participants

The initial sample was composed of 87 participants including 39 adults (25 women; mean ± SD age: 23,90 ± 2,12 years; range, 20–30 years) and 48 children (24 girls; mean ± SD age: 9,67 ± 1,78 years; range, 7–12 years). All participants and legally authorized representatives for participants age below 18 gave written informed consent before their inclusion in this study approved by the local Biomedical Ethics Committee [CUB Hôpital Erasme - Université Libre de Bruxelles (ULB, 018/2016)]. All participants were native French speakers. They had no medication or neurological, learning or language disabilities or developmental delay history.

Participants were asked to respect their usual sleep habits at least the 2 nights preceding the experiment. Sleep quality and sleep habits over the past month were assessed using the Pittsburgh Sleep Quality Index (PSQI)^57^ for adults, or the Sleep Disturbances Scale for Children (SDSC)^58^ for children. To estimate the maintenance of the sleep habits during at least two nights prior the experimental night and during the experiment, sleep duration and latency were assessed using the St. Mary’s Hospital Sleep Questionnaire^59^. As retrieval sessions occurred at different times of the day depending on the experimental condition, circadian chronotypes and vigilance states were carefully controlled to avoid any time-of-the-day effect or vigilance variation on retention performance. Circadian chronotype was assessed with the Morningness-Eveningness Questionnaire in adults (MEQ)^60^ and with the Children Morningness-Eveningness Preference in children (CMEP)^61^. Participants with an extreme circadian chronotype were excluded.

Vigilance state was assessed at each retrieval (delayed and immediate) session using the Psychomotor Vigilance Task (PVT, 5 minutes-duration version)^62^, according to standards in the field^63^. Participants were asked to press a button as fast as possible each time a digital counter started, with the reaction time as a dependent measure. The PVT-task is a gold standard measure to detect changes in vigilance related to circadian variations^62–64^. The PVT is also routinely used in sleep and memory studies to control for a possible impact of variation in vigilance state on learning and performance measures^8,29,63–67^. To control for such confound in our protocol, we administered the PVT to our participants both in the morning and the evening sessions.

The final sample was composed of 64 healthy subjects after exclusion of participants with (i) abnormal sleep habits, quantity or quality before or during the experimental night, (ii) extreme circadian chronotype, (iii) significant difference of vigilance between retrieval sessions. The final sample included 34 adults (21 women; mean ± SD age: 23.8 ± 2,22 years; range, 20–30 years) and 30 children (17 girls; mean ± SD age: 9.7 ± 1,77 years; range, 7–12 years). All participants were randomly assigned either to a sleep [Sleep, 15 children (mean ± SD age: 9.47 ± 1,64 years; 8 girls); 16 adults (mean ± SD age: 23.0 ± 2,39 years; 10 women)] or a wake [Wake, 15 children (mean ± SD age: 9,87 ± 1,92 years; 10 girls); 18 adults (mean ± SD age: 24,5 ± 1,86 years; 11 women)] condition. Adults received a financial compensation while children received a gift voucher for their participation in the study.

### Materials

**Figure 3:**
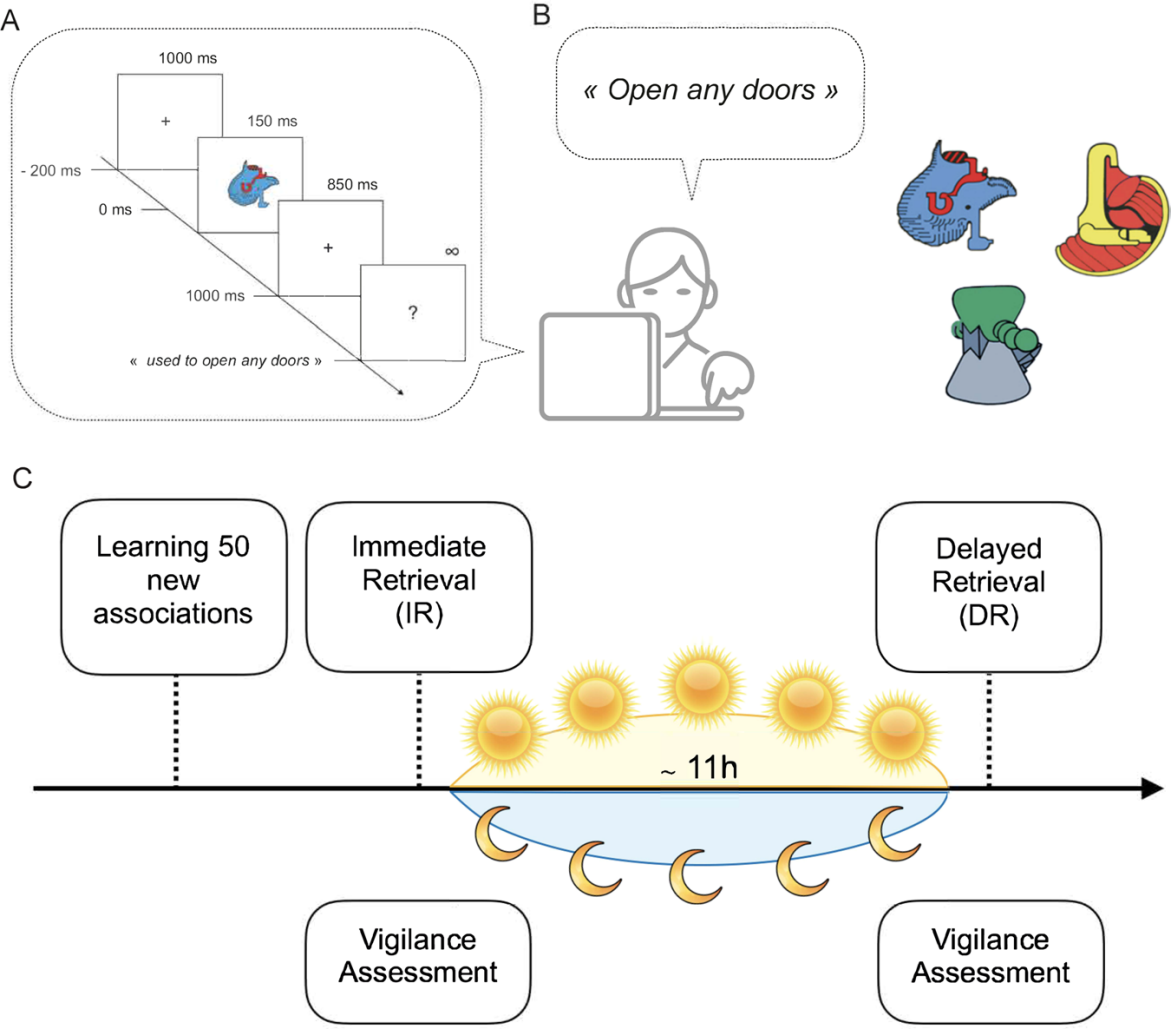
Experimental task and procedure. (**A**) Picture definition task: at each session, children and adults were asked to provide the definition of the non-object presented on the screen. Responses had to be given after the appearance of the question mark (1 s after stimulus onset). (**B**) Sample illustrations of the 50 non-objects used. (**C**) Experimental protocol: children and adults had to learn the definition of the 50 non-objects presented in the morning (Wake condition) or in the evening (Sleep condition) and directly retrieve it during the immediate retrieval session. Psychomotor vigilance was also assessed using the 5-minutes of the PVT. After a 10–12-h retention interval filled with sleep (children, N =15 ; adults, N = 16) or wakefulness (children, N = 15; adults, N = 18), a delayed retrieval of the 50 magical functions associated to the non-objects occurred, followed by the 5-minutes psychomotor vigilance task.

One hundred colored 2D outline drawings of unfamiliar non-objects (see Fig. 3A) created by the same artist and paired using PHOTOSHOP 2 in terms of size (6×6 cm on screen), intensity, colors and contrast were used. Non-objects were directly adapted from two existing databases^68^. All the NO were presented on the same 17 inch screen laptop for all participants, at a distance of 80 cm of the eyes, with a 4°x4° visual angle. The idiosyncratic character of the to-be-learned material was confirmed by a pre-test performed with a separate group of children (N = 37) to ensure that none of those non-objects were associated with any meaning. Each of the non-objects was randomly associated with a definition of the object’s magical (imaginary) function (e.g., With this object you can “open any doors”, “see through the walls”, “stop the rain”, “quickly heal wounds”, “read people’s thoughts”). All definitions were in French and were 4 to 7 words long. Four lists of 50 to-be-learned stimuli (randomly selected from the set of 100 non-objects) were created in counterbalanced order. One list was assigned to each participant at the learning session (see Fig. 3B). A complete description of the properties of the material and learning task can be found in Urbain *et al*. (2013a, 2016).

### Learning and Retrieval sessions and Experimental procedure

All participants were tested at home during 3 sessions: a learning session, an Immediate Retrieval (IR) session and a Delayed Retrieval (DR) session. All sessions were conducted in a quiet environment. In the Sleep condition, the learning and IR sessions occurred in the evening while the DR session occurred in the morning after a night of sleep. In the Wake condition, learning and IR took place in the morning, and DR in the evening without a sleep interval (see Fig. 3C).

During the learning session, all participants learned the 50 “magical” functions of each non-object. The learning session included ten learning blocks during which each participant learned five non-objects. For each learning trial, a non-object was presented on the computer screen by the experimenter who mentioned its magical function aloud to the participant. Then, each non-object was presented during 150 ms followed by 850 ms of a white screen and finally a question mark indicating to the participant to repeat the function they had just been taught (see Fig. 3B). After each five non-objects, a recapitulative test (including five non-objects) was administered. Feedback with correct responses was given to the participant during this five-by-five learning session but not during the IR or DR session (see below; a detailed description of the learning procedure is provided in Urbain *et al*., 2013a).

The IR session occurred immediately after the learning session. During the IR session, the 50 non-objects were again presented one by one randomly. As for the learning session, each non-object was presented during the IR session for 150 ms, followed by 850 ms of a white screen, and then the question mark indicating to the participant to formulate the answer (the most complete definition; or “I skip” in case of forgotten items). Participants had to correctly retrieve at least 60% of the novel verbal associations during that IR session that followed the learning session. If the participant did not achieve a 60% success rate, the learning session restarted only presenting the forgotten items. Once the success criterion was reached, meaning that the participant succeeded in remembering at least 30 definitions out of 50, the IR session was completed.

The DR session occurred at home, on average eleven hours (10–12 hours) after the IR session and in exactly the same conditions except that no success criterion had to be reached. To avoid potential effects of sleep inertia, the DR session in the Sleep condition and the learning Session in the Wake condition occurred one hour after participants woke up.

### Data acquisition and analyses

Retrieval performance at IR and DR was encoded using a computer program (MATLAB 6.1 R12.1, Mathworks, Sherbom, MA, 2004). Additionally, participants’ oral responses were recorded for qualitative purpose. Statistical analyses were conducted using STATISTICA 12 software (TIBCO SOFTWARE, California, USA, 2016).

Declarative memory retention performance was computed using a retention index (%), subtracting the percentage of correct responses at DR from the percentage of correct responses at IR for each participant. Analyses were then conducted using a factorial ANOVA on the retention indices with two between-subject factors AGE GROUP (Children vs. Adults) and CONDITION (Sleep vs. Wake). Post hoc Tuckey tests were used to decompose ANOVA effects. Bayesian t-tests analyses were also conducted to provide further evidence in favor of either the null or the experimental hypothesis. Bayes Factors (BF) are interpretable as an odds ratio and a default mode for Bayesian t-test^69^. A BF value less than 1/3 is viewed as strong supportive evidence for the null hypothesis (i.e., no difference between groups) whereas BF values > 3 strongly support the experimental hypothesis of between-group differences. An intermediate BF value (between 1/3 and 3) is viewed as inconclusive.

To ensure that memory performance at immediate and delayed retrieval sessions was not confounded by a circadian effect in both conditions (Sleep: evening-morning vs. Wake: morning-evening) or vigilance state, vigilance was assessed at each retrieval (delayed vs. immediate) session using the PVT task. Analyses consisted of a repeated measure ANOVA on PVT mean reaction times (RTs) with a within-subjects factor SESSION (IR vs. DR) and two between-subjects factors AGE GROUP (Children vs. Adults) and CONDITION (Sleep vs. Wake).

Student’s t-tests for independent samples (or if variance were unequal, Welch’s t-tests) were computed to assess potential differences between groups or conditions on sleep parameters or the number of trials needed to reach the successful learning criterion. In the Sleep condition, correlational analyses were conducted between sleep onset or sleep duration on the experiment night (N0) and memory retention indices.

The significance level was set at p < 0.05.
