# Author Correction: The power of children’s sleep - Improved declarative memory consolidation in children compared with adults

**DOI:** 10.1038/s41598-020-69655-y

**Published:** 2020-07-24

**Authors:** Anna Peiffer, Maud Brichet, Xavier De Tiège, Philippe Peigneux, Charline Urbain

**Affiliations:** 1 Laboratoire de Cartographie fonctionnelle du Cerveau (LCFC), UNI – ULB Neuroscience Institute, Université libre de Bruxelles (ULB), Brussels, Belgium; 2 Neuropsychology and Functional Imaging Research Group (UR2NF), Center for Research in Cognition and Neurosciences (CRCN), UNI – ULB Neuroscience Institute, Université libre de Bruxelles (ULB), Brussels, Belgium

Correction to: *Scientific Reports* https://doi.org/10.1038/s41598-020-66880-3, published online 19 June 2020

This Article contains errors. The Acknowledgements section in this Article is incomplete.

“This work was supported by a grant of the Fonds de la Recherche Scientifique (FRS-FNRS, Belgium: CREDIT DE RECHERCHE: n° 29149840) - project “The role of slow sleep oscillations and associated brain connectivity mechanisms in memory consolidation and executive functions”. We would also like to thank Prof M. J. Taylor for her precious help in proofreading the manuscript.”

should read:

"This work was supported by two grants from the Fonds de la Recherche Scientifique (FRS-FNRS, Belgium: CREDIT DE RECHERCHE: n° 29149840 and FRS-FWO Excellence Of Science (EOS) MEMODYN: 30446199). We also thank Dr M. J. Taylor for her precious help in proofreading the manuscript."

Additionally, the Article contains errors in Table 2 where the value for Vigilance Index of children in the wake condition should read -0.2 ± 30.32.

This Article also contains a typographical error in the Introduction section where,

“According to system consolidation theories, declarative knowledgeis progressively integrated into long-term memory through brain plasticity processes generating functional and structural changes at the neural level^1,2^.”

should read:

“According to system consolidation theories, declarative knowledge is progressively integrated into long-term memory through brain plasticity processes generating functional and structural changes at the neural level^1,2^.”

